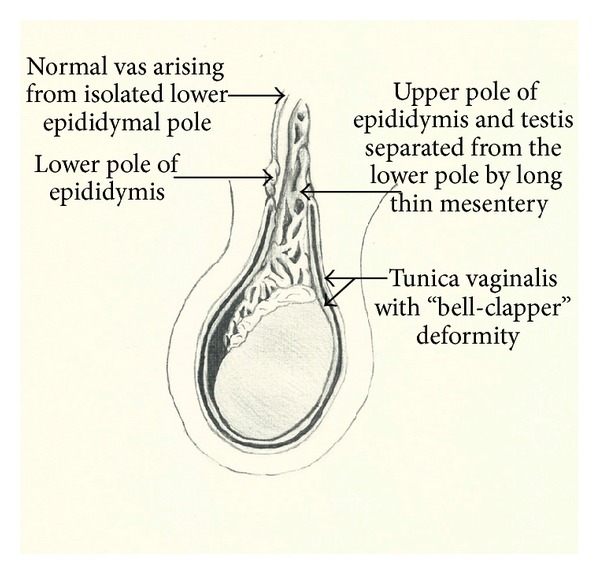# Epididymal Polar Dissociation: A Previously Undescribed Anatomical Variant

**DOI:** 10.1155/2014/360436

**Published:** 2014-05-04

**Authors:** Stephen McCain, Scott McCain, David Mark, Robin Brown

**Affiliations:** Daisy Hill Hospital, 5 Hospital Road, Newry BT35 8DR, UK

## Abstract

The normal male external genitalia include the testicles with the epididymis attached posteriorly and the vas deferens arising from this. This case describes an anatomical variation of this normal anatomy not previously reported in the literature. A 17-year-old boy presented with symptoms of intermittent testicular torsion and underwent scrotal exploration. On the left side there was a bell-clapper deformity with the epididymis separated into two parts with the lower pole high in the scrotum and attached to the tunica vaginalis. A normal vas deferens was seen to arise from the isolated lower pole of the epididymis. There was no connection between the vas deferens and the testis or upper pole of epidiymis. This case reminds us of the possibility of anatomical variations and the importance of keeping them in mind to prevent complications at time of surgery.

## 1. Introduction

An understanding of the anatomy of the male external genitalia, namely, the scrotum, testicles, epididymis, and cord structures, is crucial during the exploration of the testis in cases of suspected torsion. Awareness of the possible variations that may exist is necessary for appropriate surgical management and the prevention of complications during this and other urology operations. There is a recognised increased risk of complications in procedures with anatomical variations [1, 2].

This case report reveals an anatomical variant of the epididymis and vas deferens not previously described in the literature. There is also a brief discussion of the normal anatomy and some complications that have been described up until now.

## 2. Case Presentation

A 17-year-old boy was referred complaining of intermittent left testicular pain for one year. He described the pain as sudden in onset, with each episode lasting several hours in duration. Associated symptoms included nausea and abnormal position of his left testicle. He was asymptomatic on the right side. There were no developmental problems or history of testicular trauma. Ultrasound of scrotum revealed an incidental 2.3 cm epididymal cyst on the right but no abnormalities on the left.

He was diagnosed clinically with intermittent left sided testicular torsion and was admitted for exploration of his left hemiscrotum with the option of orchidopexy.

Intraoperatively there was a bell-clapper deformity and an anatomical abnormality was identified. The epididymis was separated into two parts with the lower pole high in the scrotum and attached to the tunica vaginalis. The upper pole and testis were separated from the lower pole by a long thin mesentery, 6-7 cm in length and containing the testicular vessels. A normal vas deferens was seen to arise from the isolated lower pole of the epididymis. There was no connection between the vas deferens and the testis or upper pole of epidiymis. Left orchidopexy was performed. Exploration of the right side revealed no abnormality. The operative findings are depicted in Figure 1.

Postoperative intravenous urogram excluded any other congenital renal tract abnormalities.

At 6-month followup, he was asymptomatic.

## 3. Discussion

The male external genitalia include the scrotum which contains the testicles with the epididymis lying along the posterior border of the testicle. The vas deferens is the excretory duct of the testicle and commences at the lower end of the epididymis. It then ascends along the posterior border of the testis and medial side of the epididymis before travelling in the spermatic cord along with the blood and nerve supply to the testicle and epididymis. It divides from the spermatic cord at the deep inguinal ring extending downwards and backwards towards the seminal vesicles. Many anatomical variations and abnormalities have been reported [1–3]. More than one anomaly may be present and these can have implications for fertility [4].

The epididymis is normally attached to the posterior aspect of the testis at the epididymal head and tail. There are six reported anatomical variations (Turek et al.): Type I—the epididymis that is united with the testis by its head and tail; Type II—the epididymis that is totally united with the testis; Type III—disjunction of the epididymal tail; Type IV—disjunction of epididymal head; Type V—total disjunction between the epididymis and the testis; and Type VI—epididymal atresia [5]. Kuçukaydin et al. proposed a modification to this classification by changing Type I to 1a which is the normal anatomy and 1b which is an elongated loop-like epididymis [4]. The anatomical variation that we report in our case has not been previously reported and does not fit into any of the six variations described. This is the first case that reports the presence of a normal vas deferens attached to the lower pole of the epididymis with no association with the testicle.

A bell-clapper deformity occurs when the tunica vaginalis is connected higher than normal on the testicle causing excessive testicular mobility [6]. The mesorchium joins the testis to the epididymis and can be excessively long in cases of epididymal disjunction or elongated epididymis. Both of these anatomical variants are associated with a higher incidence of testicular torsion than in normal anatomy. A combination of these was identified in this patient. These conditions are more common in patients with cryptorchidism but this was not present in this case [7].

Favorito et al. analysed the anatomical anomalies of the epididymis and tunica vaginalis in 25 patients with testicular torsion. Variants of epididymal anatomy were graded as found in the literature and classified into 6 groups as those described above. They found that the bell-clapper was the most frequent cause of torsion, and the most frequently found anatomical relation between the testis and epididymis in the study group was Type I as described above [7].

This case is the first to be reported in which a normal vas deferens is present but not connected to the testicle. Ductal abnormalities are reported in the literature and have an incidence of 10 to 27 percent in infertile patients compared to 0.5 to 1 percent in the normal population [8]. Bilateral absence of the vas deferens is the most common abnormality and occurs in 1-2% of men presenting with infertility. Males with absence of the vas deferens commonly have other urogenital anomalies [9].

Variations in testicular anatomy have also been reported including cryptorchidism, polyorchidism, monorchidism, and testicular agenesis. Anatomical abnormalities of the vas deferens and epididymis are reported in many studies of cryptorchidism. Cryptorchidism, the absence of one or both testes from the scrotum, is the most common birth defect of the male genitalia and occurs in approximately 1.5% of male infants [10]. Mollaeian et al. studied the prevalence of epididymal and ductal anomalies associated with cryptorchidism. 652 patients who underwent surgical intervention for management of undescended testicles were examined intraoperatively. Epididymal and ductal anomalies occurred in 36 percent of cases (235 of 652 cases), with flimsy attachment of the head of epididymis to the testes the most common anomaly [11].

This case reminds us of the anatomical abnormalities that can occur within the male scrotum. Caution should be exercised during scrotal exploration to ensure early recognition of epididymal abnormalities and prevent inadvertent injury with its subsequent consequences on fertility. It is important to rule out coexisting urogenital abnormalities. Furthermore, it is important to recognise intermittent testicular torsion as a separate entity from acute torsion and manage it accordingly.

## Figures and Tables

**Figure 1:**